# SlideRing: Robust Dual-IMU Thumb-to-Finger Text Input for Virtual Reality

**DOI:** 10.3390/s26134210

**Published:** 2026-07-03

**Authors:** Tao Sun, Nuo Jia, Dawei Jiao

**Affiliations:** 1 Changchun Institute of Optics, Fine Mechanics and Physics, Chinese Academy of Sciences, Changchun 130033, China; 2 College of Computer Science and Technology, Jilin University, Changchun 130012, China; 3 China Petroleum Jilin Chemical Engineering Co., Ltd., Jilin 132000, China

**Keywords:** virtual reality, text entry, inertial measurement unit, micro-gestures, thumb-to-finger interaction, wearable input

## Abstract

Text entry remains a bottleneck for productivity-oriented Virtual Reality (VR), especially in scenarios where optical hand tracking is unstable because of self-occlusion, poor lighting, or out-of-view interaction. We present SlideRing, a dual-thumb wearable text-entry method that senses thumb-to-finger micro-gestures with two miniature Inertial Measurement Units (IMUs). SlideRing defines a 30-command interaction space from two hands, three target fingers, and five gesture types, then maps these commands to a full alphabetic keyboard through two complementary strategies: an ergonomic layout optimized for low movement cost and a QWERTY-compatible layout optimized for learnability. To decode subtle inertial signals, we design a dual-stream recognition model with a Statistical Feature Encoder, a Temporal Feature Encoder, and a context-aware gating module for joint finger–action classification. In offline evaluation, the model reaches 96.5% target-finger accuracy and 94.2% action-type accuracy. In a five-day text-entry study, the ergonomic layout improves from 7.43 to 15.75 words per minute (WPM), while the QWERTY-compatible layout improves from 10.55 to 15.25 WPM. The ergonomic layout reduces physical demand, whereas the QWERTY-compatible layout lowers initial mental load. These results suggest that IMU-based thumb-to-finger input has the potential to provide robust, low-visual-demand text entry for constrained VR environments.

## 1. Introduction

As Virtual Reality (VR) expands from entertainment to mobile work, remote collaboration, and productivity-oriented spatial computing, text entry becomes a fundamental interaction primitive. Users must enter messages, search queries, passwords, notes, and longer text without breaking immersion, yet VR input remains difficult because speed, accuracy, physical comfort, portability, and visual attention must all be balanced at once. Existing approaches based on physical keyboards, touchscreens, and wearable gloves can improve familiarity or sensing quality, but they increase setup cost and reduce mobility [1,2,3,4,5]. Other techniques based on gaze, speech, and mid-air interaction reduce hardware dependence, but often introduce new problems such as social awkwardness, sensitivity to noise, eye fatigue, or prolonged arm fatigue [6,7,8,9,10]. Consequently, a practical VR text-entry method should remain lightweight while still supporting stable and efficient input in everyday use.

Among existing designs, compact and thumb-to-finger input methods are especially relevant because they reduce visual footprint and exploit the hand itself as an input surface. Ambiguous or compressed layouts such as FingerT9, Force9, T18, and EyeClick reduce occupied interaction space, while alternative topologies such as WrisText and HiPad attempt to preserve efficiency under limited display area [11,12,13,14,15,16]. Prior thumb-to-finger systems further show that tactile and proprioceptive cues from the hand can support low-visual-demand text entry [17,18,19,20,21,22]. However, these methods still reveal a major sensing tradeoff. Vision-based solutions are lightweight and hardware-free, but they are vulnerable to self-occlusion, unstable illumination, and out-of-view interaction. Sensorized solutions are often more robust, yet many require multiple sensors distributed across the hand, depend on an external surface, or prioritize simple command vocabularies instead of practical full-alphabet typing.

These limitations become most visible in constrained VR scenarios, where users lower their hands, rest near the torso, or type outside the reliable field of view of headset cameras. In such cases, inertial sensing is attractive because it captures local motion, impact signatures, and orientation changes directly at the interaction site. Prior Inertial Measurement Unit (IMU)-based systems such as QwertyRing, AccurateandLow-Latency, DualRing, and DRG-Keyboard demonstrate that finger-worn inertial sensors can reliably sense subtle touch and gesture events [23,24,25,26]. Building on this direction, we propose SlideRing, a dual-thumb IMU-based text-entry technique that maps thumb slides and taps on the index, middle, and ring fingers to a full alphabetic command space. SlideRing combines two complementary keyboard layouts—an ergonomic layout optimized for movement economy and a QWERTY-compatible layout optimized for learnability—with a dual-stream recognition network that fuses statistical and temporal features for robust finger–action decoding. In this way, SlideRing is designed not as a replacement for all VR text-entry methods, but as a robust complement for situations in which vision-driven approaches become unreliable or overly demanding.

The main contributions of this work are as follows:

- We define a dual-hand thumb-to-finger micro-gesture space for VR text entry and characterize its biomechanical cost structure.
- We propose a dual-stream inertial recognition model with context-aware gating for joint finger and action decoding, reaching 96.5% target-finger accuracy and 94.2% action-type accuracy.
- We introduce two full-alphabet layouts for the same gesture space, one optimized for movement economy and one optimized for QWERTY familiarity.
- We report offline recognition, five-day learning curves, and subjective workload results, showing that SlideRing complements vision-based text entry in constrained environments.

## 2. Related Work

### 2.1. Text Entry in Head-Mounted Displays

Text entry is one of the most important interaction tasks in Head-Mounted Displays (HMDs). Existing research has explored physical-device-assisted input, head- or gaze-based input, speech input, and gesture-based input. Physical keyboards, controllers, and touchscreens remain attractive because they are familiar and can provide reliable tactile feedback [2,3,16]. Head- and gaze-based techniques further reduce manual effort and can be effective when display space is limited [7,14,27,28]. Speech interaction is also appealing because it is hands-free, but its usefulness is often constrained by environmental noise and privacy concerns [29]. In commercial VR systems, mainstream text entry still commonly relies on ray-casting on floating virtual keyboards or pinch-style key selection. Although effective for occasional input, such approaches often require users to devote substantial visual attention to the keyboard and may suffer from target occlusion or reduced precision when the interface is aggressively compacted.

To alleviate limited display space, many studies have introduced multi-letter mapping and keyboard compression. FingerT9 maps T9-style input onto finger segments to move the interaction region away from the screen [11]. Force9 exploits multiple pressure levels on smartwatch touchscreens to increase the number of effective input states without enlarging the visible keyboard [12]. PinchType and T18 preserve part of the QWERTY structure by merging keys into larger ambiguous groups and decoding the intended output with language models or constrained candidate sets [13,18]. Other work reshapes the keyboard topology itself. WrisText uses wrist gestures on a watch-like device, whereas HiPad employs a circular touchpad-matched layout for VR controllers [15,16]. These methods successfully reduce visual footprint, but they often introduce higher cognitive load, additional disambiguation burden, or repetitive movement patterns that become tiring in prolonged text-entry tasks. Prior work on spatial interaction further shows that unstable pointing and selection can trigger the Heisenberg effect, and that reducing musculoskeletal strain is essential for sustaining performance in immersive environments [9,10].

### 2.2. Text Entry Based on Hand Pose and Wearable Sensing

Hand-pose-based text entry aims to decode user intent from finger motion and contact patterns. Early systems relied heavily on data gloves and instrumented finger coverings. CyberGlove reconstructed continuous hand posture through dense bend sensing, while PeregrineGlove and related contact-based systems converted finger-to-finger touch into discrete commands [30,31,32]. Later textile and flexible-sensor systems such as Plex and TIMMi pursued lighter-weight wearable solutions for eyes-free interaction [33,34]. These approaches demonstrated that finger-level sensing can support mobile text entry, but they also highlighted persistent drawbacks such as bulk, poor breathability, and nontrivial donning cost.

Miniature IMUs provide a more compact route to hand-pose sensing and have become increasingly important in wearable text entry. LightRing showed that ring-like inertial devices can capture continuous movement on arbitrary surfaces [35]. AccurateandLow-Latency improved touch detection with a finger-worn IMU and demonstrated that inertial sensing can substantially strengthen contact inference compared with optical-only approaches [24]. QwertyRing further showed that a single IMU ring can support touch typing on physical surfaces, while DualRing and DRG-Keyboard extended the idea to dual-sensor setups for richer within-hand interaction and subtle fingertip typing [23,25,26]. Despite these advances, glove-based systems remain too cumbersome for daily use, and even lightweight multi-ring systems may interfere with natural grasping or depend on surface contact. This creates a clear need for minimal-wear sensing schemes that preserve robustness while avoiding excessive hardware intrusion.

### 2.3. Text Entry Using Unconventional Body Parts

Beyond hand-centric interaction, several researchers have explored other body regions as input surfaces. TouchEditor placed flexible piezoresistive sensors on the arm to support cursor control and text editing in speech-unfriendly settings [8]. OnArmQWERTY projected a virtual keyboard onto the arm and used optical tracking for text selection [36]. Foot-based techniques adapted typing to lower-limb gestures in VR, while FingerText compressed interaction to very small regions such as the fingernail [37,38]. These methods creatively expand the physical design space of text entry, but they often rely on uncommon motor habits, specialized hardware, or externally tracked surfaces. As a consequence, they may impose higher fatigue, stronger cognitive mismatch with everyday typing habits, or greater deployment complexity than hand-centered micro-gesture approaches.

### 2.4. Thumb-to-Finger Micro-Gesture Interaction

Thumb-to-finger micro-gesture interaction is especially promising because it combines low movement amplitude, strong proprioceptive grounding, and passive tactile feedback from skin contact. Earlier work showed that finger regions can serve as distinct tactile targets and that thumb-to-finger interaction supports subtle and eyes-free control [17]. Building on this observation, researchers explored multiple sensing strategies. DigiTouch and HiFinger used gloves or thin pressure sensors to track thumb contact across the fingers [4,5]. Quadmetric and FingerT9 mapped ambiguous letter groups to finger regions to improve compactness and mobility [11,39]. PrinType introduced fingerprint sensing on the thumb, while PinchType leveraged comfortable thumb-to-fingertip pinches and language-model disambiguation [18,20]. In parallel, vision-based systems such as ThumbAir and TipTopTyping attempted to eliminate wearables entirely through camera-based gesture recognition [19,21]. BiTipText and related fingertip-scale interfaces further compressed the interaction area to tiny capacitive zones on the finger surface [22].

Although these methods demonstrate the richness of thumb-to-finger interaction, they also reveal a persistent tradeoff between sensing robustness and interaction burden. Wearable solutions often offer better signal quality but may require multiple sensors, wires, or finger-mounted modules that reduce naturalness. Pure vision-based solutions are more lightweight, yet they remain vulnerable to self-occlusion, view-angle changes, and fine-grained spatial ambiguity when many letters must be mapped onto a very small hand area. Therefore, an important open question is how to preserve the tactile and ergonomic advantages of thumb-to-finger interaction while achieving robust recognition with minimal instrumentation. SlideRing addresses this question by using only two thumb-mounted IMUs, thereby avoiding distributed finger wearables while remaining robust in situations where camera-based methods become unreliable.

## 3. SlideRing System Design

### 3.1. Interaction Design

#### 3.1.1. Design Rationale

The design of SlideRing is motivated by a simple interaction principle: instead of asking users to perform unsupported mid-air gestures, it anchors text entry to physically supported thumb contacts on the finger surface. The central idea is to exploit passive haptic feedback generated when the thumb pad touches and slides on the phalangeal skin. This contact provides a stable mechanical guide, frictional damping, and proprioceptive confirmation, thereby reducing the need to visually verify absolute hand position during text entry.

In conventional free-space gestures, users often struggle to perceive the start point, boundary, and extent of a movement because there is no physical reference. SlideRing addresses this limitation by redefining input as discrete taps and slides performed by the thumb on the index, middle, or ring finger of the same hand. The joints and finger segments naturally act as tactile landmarks. Users can therefore sense whether the thumb is near the fingertip, near the finger root, or passing through the middle segment, which makes action amplitude easier to control without continuous visual supervision.

This physical-contact design is also important from a sensing perspective. When the thumb taps or slides over the finger surface, the interaction produces transient impacts, friction-driven vibration, and local orientation changes that propagate to the thumb-mounted inertial sensor. Compared with camera-only observation, these inertial signatures are more directly tied to the mechanical event itself. As a result, the gesture vocabulary is designed not only for user comfort, but also for generating high-discriminability sensor patterns for the downstream recognition model.

#### 3.1.2. Thumb-to-Finger Micro-Gesture Set

Based on the above rationale, SlideRing defines a micro-gesture space centered on “thumb-to-target-finger” contact. Because finger independence and movement resistance differ across the hand, the interaction targets only the index, middle, and ring fingers. The index finger is the most independent and easiest to reach, the middle finger provides moderate independence, and the ring finger is the least independent because of tendon coupling and larger biomechanical resistance when crossed by the thumb.

To keep the gesture space compact but expressive, we constrain input to two basic motion families: tapping and longitudinal sliding. Specifically, each target finger supports five discrete actions: tap, short inward slide, short outward slide, long inward slide, and long outward slide. With two hands, three target fingers per hand, and five actions per finger, the complete design exposes 30 available commands. This structured action set serves two purposes simultaneously: it limits redundant movement in the hand, and it yields inertia patterns with distinct temporal and statistical characteristics, such as sharp acceleration impulses for taps and sustained angular-velocity changes for slides.

The physical-effort ranking in Table 1 is later reused as the basis of the keyboard-mapping cost model. In other words, the interaction design, recognition algorithm, and layout optimization are intentionally coupled: the action set is chosen to be comfortable enough for repeated use, mechanically meaningful enough for inertial sensing, and structured enough to support cost-aware character assignment.

### 3.2. Micro-Gesture Recognition Algorithm

**Figure 1:**
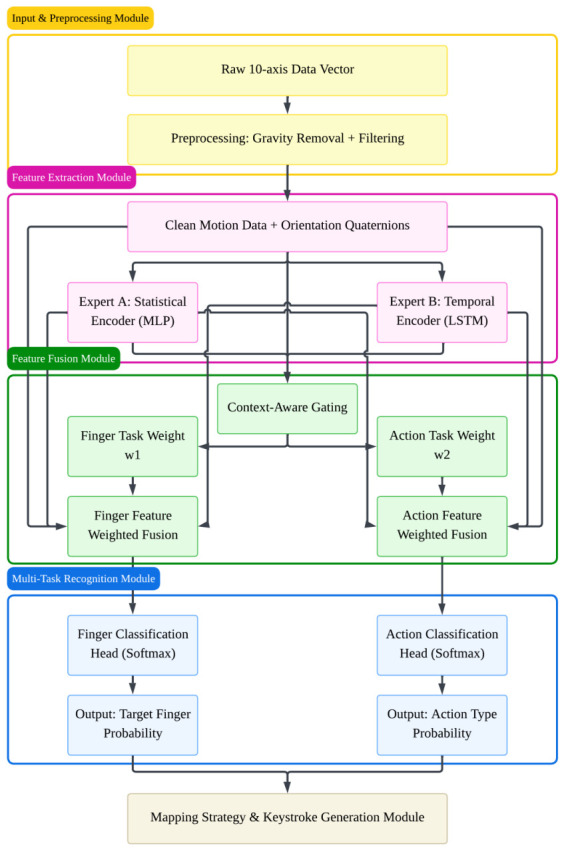
Architecture of the SlideRing multi-task micro-gesture recognition pipeline, including preprocessing, dual-stream feature extraction, context-aware fusion, and task-specific classification.

To recover user intent from noisy inertial measurements, SlideRing adopts a deep multi-task recognition pipeline organized into four functional stages: inertial preprocessing, dual-stream feature extraction, context-aware feature fusion, and multi-task recognition. As shown in Figure 1, the model is designed to jointly infer two coupled outputs from the same input window: the target finger being touched and the action type being executed. The statistical encoder summarizes posture-related cues, the temporal encoder models motion evolution, and the context-aware gating module assigns task-specific fusion weights before the finger and action classification heads.

#### 3.2.1. Inertial Preprocessing and Gravity Compensation

Each thumb-mounted sensing node streams a 10-dimensional sample consisting of tri-axial acceleration, tri-axial angular velocity, and quaternion-based orientation. Because thumb micro-gestures have small amplitude, raw acceleration is easily dominated by gravity and gross hand motion. We therefore first estimate the gravity component in the sensor frame. Let $a_{\mathrm{raw}}=\left(a_{x},a_{y},a_{z}\right)$ denote raw acceleration and $q=(q_{0},q_{1},q_{2},q_{3})$ the orientation quaternion. The projected gravity vector is computed as

(1) $$v_{g}=\left[\begin{array}{c} 2(q_{1}q_{3}-q_{0}q_{2})\\ 2(q_{0}q_{1}+q_{2}q_{3})\\ q_{0}^{2}-q_{1}^{2}-q_{2}^{2}+q_{3}^{2} \end{array}\right]g,$$

and the linear acceleration component used for recognition is obtained by

(2) $$a_{\mathrm{lin}}=a_{\mathrm{raw}}-v_{g}.$$

After gravity compensation, the continuous signal stream is reorganized into a fixed-length sliding window. This preprocessing step produces a tensorized sequence that preserves the local motion evolution of a tap or slide while suppressing nuisance variation from posture drift and static load.

#### 3.2.2. Dual-Stream Statistical and Temporal Feature Encoders

The recognition model explicitly separates the static and dynamic components of thumb micro-gestures. The Statistical Feature Encoder is designed to anchor relatively stable posture-related cues, which are useful for discriminating the contacted finger. For each window, it extracts descriptive statistics such as signal energy, kurtosis, skewness, and inter-axis correlation, then projects them into a compact representation through a multilayer perceptron. This branch serves as a high-level summary of spatial configuration and global physical state, making it especially relevant to identifying whether the thumb interacts with the index, middle, or ring finger.

In parallel, the Temporal Feature Encoder models the evolution of the inertial trace over time. This branch uses stacked Long Short-Term Memory (LSTM) layers to capture the transient structure of taps and the duration-dependent differences between short and long slides. The purpose of the temporal branch is to preserve motion continuity that would be lost if the signal were reduced to statistics alone. As a result, the temporal stream is particularly informative for distinguishing action type, especially in borderline cases where the slide direction is clear but the traveled extent is subtle.

#### 3.2.3. Context-Aware Gating and Multi-Task Decoding

Finger recognition and action recognition do not depend on the two streams equally. Therefore, SlideRing introduces a context-aware gating mechanism that adaptively fuses the static and temporal embeddings for each task. For clarity, we denote the statistical embedding from the Statistical Feature Encoder as $E_{s}$, the temporal embedding from the Temporal Feature Encoder as $E_{d}$, and the average-pooled raw-window prior as $P_{r}$. For the finger-recognition branch, $W_{f}$ and $b_{f}$ are learned gating parameters, and $\alpha_{f}$ and $\beta_{f}$ are the resulting weights assigned to the statistical and temporal streams. The finger-specific fusion weights are then computed as

(3) $$\left[\alpha_{f},\beta_{f}\right]=\mathrm{Softmax}\left(W_{f}\left[E_{s},E_{d},P_{r}\right]+b_{f}\right)$$

and the corresponding finger-task representation $V_{f}$ is formed as

(4) $$V_{f}=\mathrm{Concat}\left(\alpha_{f}E_{s}+\beta_{f}E_{d},P_{r}\right)$$

An analogous action branch uses the same compact notation with task-specific parameters and representation $V_{a}$, allowing it to favor the temporal stream more strongly when temporal cues are decisive. This design mirrors the physical prior of the task: finger identity is more related to stable pose configuration, whereas action identity is more related to temporal change. The final network therefore performs joint decoding of one target finger and one action type, providing the symbolic command that is then passed to the keyboard-mapping module.

### 3.3. Keyboard Layout Design

Once the recognition model outputs a decoded hand/finger/action command, the system must map the 30 available physical gestures to 26 alphabetic characters. Because the number of commands is only slightly larger than the size of the alphabet, layout quality directly determines both text-entry efficiency and learning difficulty. We formulate keyboard design as a separate optimization layer built on top of the recognition model. The key question is not only whether a gesture can be recognized reliably, but also whether the corresponding letter assignment minimizes cumulative effort and remains easy enough to learn in practice.

This consideration leads to two complementary design goals. The first is to maximize long-term performance by assigning frequent characters to low-fatigue gestures, thereby reducing cumulative movement cost in extended writing. The second is to reduce onboarding burden by preserving as much prior keyboard knowledge as possible. SlideRing therefore provides two layouts for the same gesture space: an ergonomic layout based on minimum fatigue cost, and a QWERTY-compatible layout based on spatial reuse of the conventional keyboard.

#### 3.3.1. Ergonomic Layout Based on Minimum Fatigue Cost

The ergonomic layout is built on the idea that frequent letters should be assigned to low-effort gestures so that long-form text entry minimizes cumulative physical work. This design directly inherits the biomechanical assumptions established in the interaction-space section. Because the index finger is easiest to access and outward long slides are most demanding, gesture assignments should not be treated as uniform. Instead, the mapping should privilege low-cost finger–action pairs for high-frequency characters.

To quantify this design principle, we define $w_{f}$ as the finger cost weight and $w_{a}$ as the action cost weight. The index, middle, and ring fingers are assigned default $w_{f}$ values of 1.0, 1.2, and 1.6, respectively, reflecting their general differences in movement independence and subjective effort. Likewise, tap is treated as the easiest action with $w_{a}=1.0$, followed by short inward slide (1.3), short outward slide (1.5), long inward slide (2.4), and long outward slide (3.0). These coefficients are not intended to represent universal biomechanical constants. Instead, they provide an initial cost model based on preliminary user-rated comfort, perceived fatigue, and the relative movement amplitude of each gesture. Because perceived fatigue differs across users, the weights are designed to be adjustable: during practice, a user-specific layout can be updated by increasing the weights of gestures that feel tiring or unstable and decreasing the weights of gestures that feel comfortable and reliable. In this way, the ergonomic layout is initialized from a common default prior but can gradually converge toward a personalized low-fatigue key arrangement.

For an arbitrary gesture combination (f,a), where *f* denotes the target finger and *a* denotes the action type, the single-use motion cost C(f,a) is defined as

(5) $$C\left(f,a\right)=w_{f}\left(f\right)w_{a}\left(a\right)$$

Given character frequencies $p_{i}$, where $p_{i}$ is the frequency of character $c_{i}$, the layout objective J(M) is

(6) $$J\left(M\right)=\sum_{i=1}^{26}p_{i}C\left(M\left(c_{i}\right)\right)$$

where $M(c_{i})$ is the gesture assigned to character $c_{i}$. This notation keeps the optimization objective compact while preserving the same cost model. In practice, this optimization is implemented with a greedy ranking strategy: gesture candidates are sorted from low to high cost, characters are sorted from high to low frequency, and the two sequences are matched in reverse order. This construction makes the ergonomic layout suitable for long-term use, because it systematically pushes high-frequency letters toward easy taps and short slides while relegating rare letters to more demanding edge gestures. Extremely costly edge combinations can also be left unused rather than forcing a full one-to-one assignment over all 30 gesture slots.

**Figure 2:**
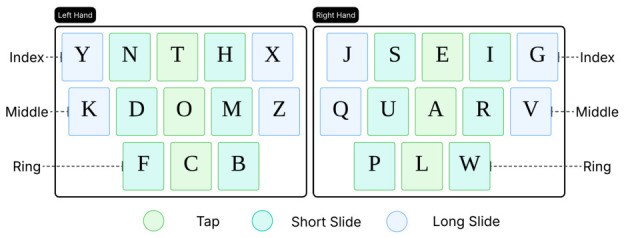
Ergonomic layout strategy: inverse matching between character frequency and gesture cost.

Figure 2 illustrates this inverse matching principle. The resulting distribution concentrates frequent letters in the low-cost zones associated with index-finger taps and short slides, while low-frequency letters are shifted toward the higher-cost periphery. As such, the ergonomic layout functions as the default efficiency-oriented mapping for extended text entry.

#### 3.3.2. QWERTY-Compatible Layout Based on Spatial Reuse

Although the ergonomic layout is favorable for long-term efficiency, it requires users to learn a new symbolic mapping. The QWERTY-compatible layout instead prioritizes immediate learnability by preserving the relative spatial logic of a standard keyboard. This mapping is formulated as a dimensionality-reduction problem: the two-dimensional QWERTY plane is decomposed into hand side, keyboard row, and horizontal offset, then re-encoded onto hand, target finger, and slide direction.

Concretely, the full alphabet is first partitioned into left-hand and right-hand regions according to the standard physical keyboard. Within each region, letters from the top row are bound to the index finger, letters from the home row to the middle finger, and letters from the bottom row to the ring finger. Horizontal displacement within the row is then represented by inward and outward slides around a local anchor action. Thus, the mapping preserves the user’s pre-existing intuition that letters in the same keyboard row should remain near one another in the new gesture space. The density-aware degradation rules used in this mapping are summarized in Table 2.

A density-aware degradation rule is used when a row segment contains fewer than five letters. In dense regions, all five actions can be used. In sparser regions, some actions are intentionally removed so that the remaining gestures are more separable and less prone to accidental triggering. This design helps the QWERTY-compatible layout maintain a balance between spatial familiarity and recognition robustness.

**Figure 3:**
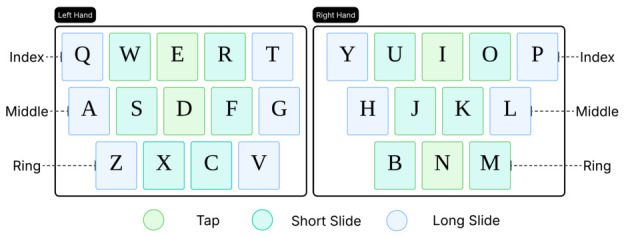
QWERTY-compatible layout: reusing the relative keyboard structure in the thumb-to-finger gesture space.

After the responsible finger is determined by keyboard row, the column position of each letter is mapped to action direction relative to a local anchor. Letters located toward the inside of the physical keyboard are assigned inward slides, whereas letters located toward the outer side are assigned outward slides; the most central and intuitive position can remain a tap when the local density permits. Figure 3 illustrates this logic in a compact visual form.

As a result, the QWERTY-compatible layout offers a low-barrier entry mode for novice users by preserving directional and row-based keyboard memory, while the ergonomic layout remains the better candidate for sustained high-frequency text entry after practice. Together, the two layouts allow SlideRing to support both rapid onboarding and long-term efficiency within the same gesture vocabulary.

## 4. Materials and Methods

### 4.1. Apparatus and Data Collection

The sensing hardware consisted of two XIAO nRF52840 Sense boards (Seeed Studio, Shenzhen, China) worn on the proximal phalanges of the left and right thumbs. Each board integrates a compact inertial sensing module, a low-power wireless communication unit, and an embedded controller in a form factor small enough for thumb-mounted deployment. In the SlideRing prototype, the boards were used as miniature wearable sensing nodes that continuously captured tri-axial acceleration and tri-axial angular velocity at 100 Hz while streaming data to the host computer through Bluetooth Low Energy (BLE).

**Figure 4:**
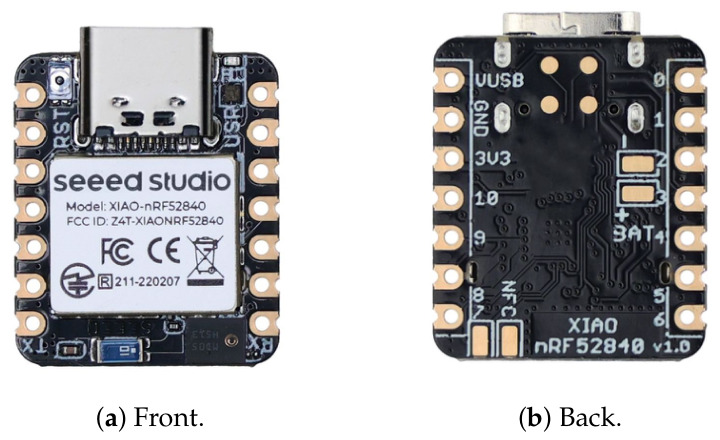
Front and back views of the thumb-mounted sensing board used in SlideRing.

As shown in Figure 4, the front and back views of the XIAO nRF52840 Sense board illustrate the compact sensing unit used in SlideRing.

A Unity-based acquisition program (Unity 2022.3.8f1c1, Unity Technologies, San Francisco, CA, USA) displayed prompted gestures and recorded synchronized sensor streams on a laptop computer equipped with an AMD Ryzen 7 4800H CPU, 16 GB RAM, and an NVIDIA RTX 2060 GPU (Nvidia, Santa Clara, CA, USA). During online interaction, the recognized characters were forwarded to a Meta Quest 2 headset (Meta, Menlo Park, CA, USA).

**Figure 5:**
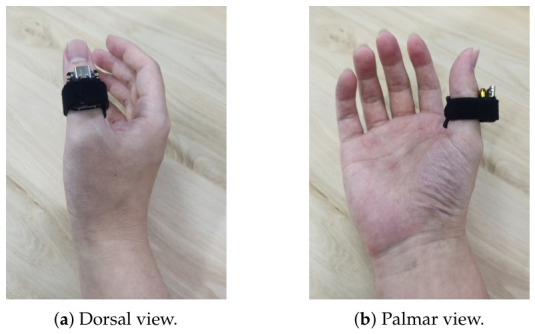
Wearing configuration of the SlideRing prototype on the thumbs.

Twelve university students (8 male, 4 female; age 22–28 years) participated in the study. During the formal data-collection stage, participants sat in a relaxed posture and wore the packaged IMU sensors on the proximal phalanges of both thumbs, as shown in Figure 5. For offline model training, each participant completed 15 gesture combinations per hand (three fingers by five actions), each repeated 12 times, yielding 4320 valid samples in total. The dataset was split into training, validation, and test sets with an 8:1:1 ratio, and we additionally report Leave-One-Subject-Out Cross-Validation (LOSO-CV) to assess user generalization.

### 4.2. Online Text-Entry Study

We evaluated SlideRing in a five-day longitudinal text-entry study. Participants transcribed phrases from MacKenzie’s phrase set [40] using both the ergonomic and QWERTY-compatible layouts. Each day consisted of 30 min of practice and evaluation. We recorded words per minute (WPM), total error rate (TER), and raw National Aeronautics and Space Administration Task Load Index (NASA-TLX) ratings [41]. WPM was computed using the standard formulation [42]:

The recognition model evaluated in this study focused on the 30 contact-based finger–action commands used for alphabetic input. During the text-entry experiment, three auxiliary non-contact thumb gestures were used for basic control operations outside the trained contact-gesture classifier: an inward air swipe of the left thumb inserted a Space, an inward air swipe of the right thumb triggered Backspace/Delete, and an outward air swipe of the right thumb served as Enter/Confirm to advance to the next prompted phrase.

(7) $$\mathrm{WPM}=\frac{|T|-1}{S}\times 60\times \frac{1}{5},$$

where |T| is the number of characters in the transcribed text and *S* is the elapsed input time in seconds.

In addition to speed, we report TER as a process-aware error metric grounded in the standard text-entry evaluation literature [43]. Unlike final-text-only error measures, TER accounts for both corrected and uncorrected mistakes during the full entry process. Following the character-stream error taxonomy introduced by Soukoreff and MacKenzie [43], let *C* denote correct characters, INF denote incorrect-not-fixed errors that remain in the final transcription, and IF denote incorrect-but-fixed errors that were produced and later corrected by the user. TER is then defined as

(8) $$\mathrm{TER}=\frac{INF+IF}{C+INF+IF}\times 100\%.$$

This metric better reflects the underlying robustness of the recognition pipeline under realistic use, because it captures not only residual output errors but also the hidden correction burden imposed on the user during entry, which is often overlooked by final-text-only measures [43]. In addition to entry performance, participants completed NASA-TLX after the final evaluation block to report perceived mental demand, physical demand, effort, and frustration.

## 5. Results

### 5.1. Offline Recognition Performance

The experimental results show that the proposed dual-stream fusion network achieves strong recognition performance. Specifically, the model reached 96.5% accuracy for target-finger classification and 94.2% accuracy for action-type classification. A complete micro-gesture command is correctly decoded only when both the target finger and action type are correctly recognized; therefore, command-level correctness is bounded by the two component decisions. These results indicate that the statistical–temporal dual-stream encoder and the context-aware gating mechanism can robustly support finger–action decoding, while the action-type branch remains the main constraint on complete command recognition.

**Figure 6:**
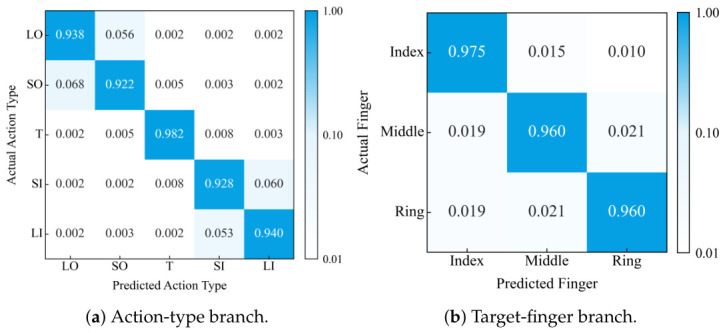
Confusion matrices for the two output branches of the SlideRing multi-task recognition model. LO, SO, T, SI, and LI denote Long Outward Slide, Short Outward Slide, Tap, Short Inward Slide, and Long Inward Slide, respectively.

To examine the error structure of the recognition model in more detail, Figure 6 shows the confusion matrices for the two output branches of the multi-task model.

Two characteristic patterns can be observed from the action-type matrix in Figure 6a. First, tap achieves the highest recognition accuracy, because thumb tapping produces a high-energy transient acceleration impulse that is highly distinctive in both the temporal and statistical streams. Second, the remaining errors are concentrated between short and long slides in the same direction. This is consistent with individual variability in how users executed movement extent, especially near the physical boundary between short and long sliding motions.

For target-finger classification, Figure 6b shows that only a very small amount of cross-finger confusion remains between the middle and ring fingers. This pattern is consistent with the biomechanical coupling of adjacent fingers: when the ring finger acts as the contact base for thumb sliding, a small resonance-like motion can also be induced in the middle finger. Despite these rare boundary cases, the overall recognition accuracy is sufficient to support the downstream text-entry task.

### 5.2. Text-Entry Performance and Error Rates

To compare the practical performance of the two keyboard layouts in realistic typing tasks, we conducted a text-entry study in which participants transcribed prompted phrases from MacKenzie’s phrase set in VR [40]. The evaluation focused on two core metrics: words per minute (WPM) and total error rate (TER). As noted in Section 4, TER combines corrected errors and uncorrected final errors, and therefore offers a more complete view of recognition robustness and user burden during actual text entry.

Table 3 summarizes the longitudinal WPM and TER results across the five practice days, complementing the learning curves with exact mean and standard-deviation values.

The tabulated values show two complementary trends. The QWERTY-compatible layout provides a clear initial advantage, with higher WPM and lower TER on Day 1, reflecting the benefit of familiar keyboard associations during early use. In contrast, the ergonomic layout shows a steeper improvement across practice sessions: its WPM increases from 7.43 to 15.75, while its TER decreases from 13.42% to 6.14%. This pattern suggests that the ergonomic mapping imposes a higher short-term learning cost but gradually benefits from its lower-effort gesture allocation. Therefore, the following learning-curve analysis separates input speed and error rate to examine how these two aspects evolve over time.

After five consecutive days of use, with 30 min of practice and evaluation per day, we obtained a full set of longitudinal learning data for both layouts. To examine the effects of layout strategy and practice time, we analyzed WPM and TER separately using two-way repeated-measures ANOVA with layout and day as within-subject factors. The results are presented separately for input speed and error rate.

**Figure 7:**
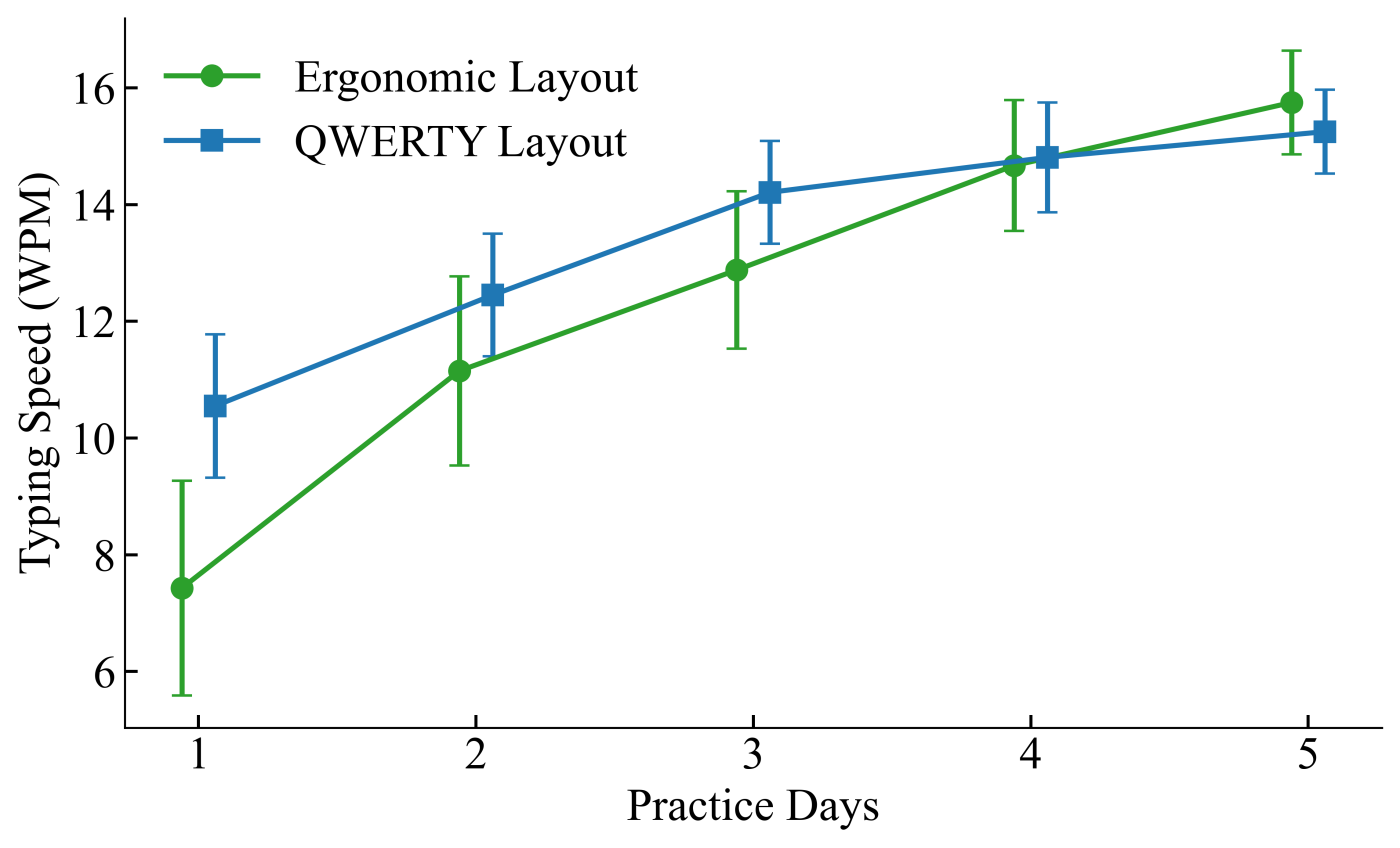
Learning curve of input speed in words per minute (WPM) for the ergonomic and QWERTY-compatible SlideRing layouts.

Figure 7 shows the evolution of input speed across the five practice days.

As shown in Figure 7, the two layouts exhibit markedly different early-stage performance. The ANOVA results show a highly significant interaction between layout and practice day for WPM ($F_{4,44}=21.35$, p<0.001). Pairwise comparisons further indicate that on Day 1, the QWERTY-compatible layout yields a significantly higher initial entry speed (M=10.55, SD=1.23) than the ergonomic layout (M=7.43, SD=1.84; t(11)=5.82, p<0.001). This advantage is consistent with the design goal of the QWERTY-compatible mapping: it reactivates users’ long-term spatial and motor memory of a conventional keyboard, allowing them to locate characters more intuitively and transfer familiar two-dimensional keyboard knowledge into the new micro-gesture space with relatively low cognitive overhead.

However, both layouts improve substantially with continued practice. By Day 5, the QWERTY-compatible layout reaches 15.25 WPM, whereas the ergonomic layout further improves to 15.75 WPM, and statistical comparison of the Day 5 results shows that the ergonomic layout now holds a significant speed advantage (t(11)=2.45, p<0.05). This crossover strongly supports the long-term benefit of the ergonomic mapping. By assigning high-frequency characters to short-span, low-fatigue motions, the ergonomic layout reduces cumulative thumb travel and muscular effort during repeated input, and therefore releases greater performance potential once the user has adapted to the mapping. Taken together, these results suggest that although the ergonomic layout imposes a higher learning cost at the beginning, it is better suited to sustained high-frequency text entry in immersive work scenarios, whereas the QWERTY-compatible layout remains particularly effective for rapid onboarding.

**Figure 8:**
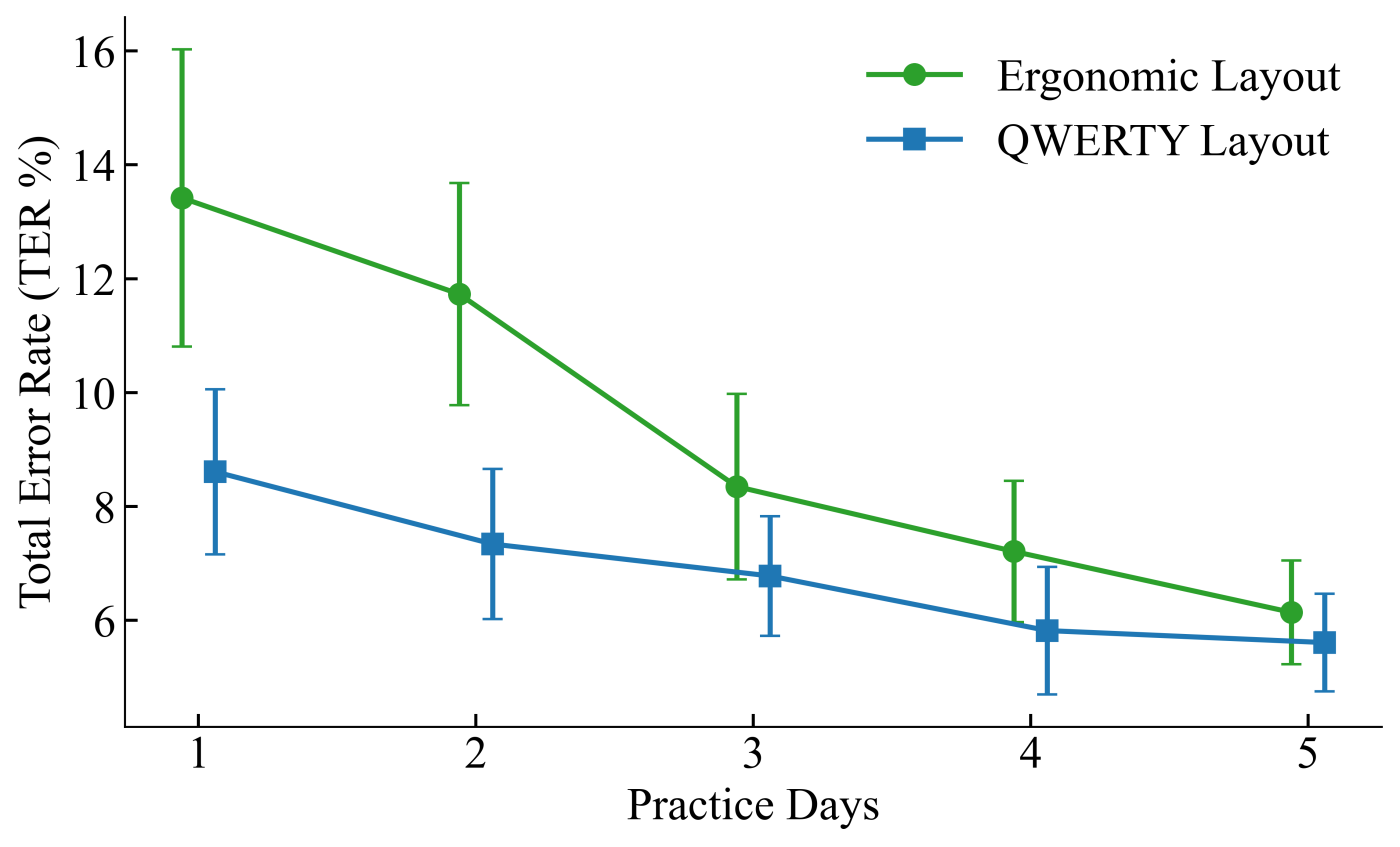
Learning curve of total error rate (TER) for the ergonomic and QWERTY-compatible SlideRing layouts.

Figure 8 shows the corresponding TER trend over the same five-day period.

The TER results show a closely related adaptation pattern. As illustrated in Figure 8, the ergonomic layout begins with a relatively high initial error rate on Day 1 (M=13.42%), significantly worse than the QWERTY-compatible layout (M=8.61%; t(11)=6.14, p<0.001). This result is consistent with the higher cognitive burden introduced when users must temporarily break away from entrenched QWERTY associations and learn a new movement-to-character mapping.

With continued practice, however, the TER of the ergonomic layout decreases rapidly and reaches 6.14% by Day 5. At that point, its difference from the QWERTY-compatible layout (5.61%) is no longer statistically significant (t(11)=1.88, p=0.087). This downward trend indicates that as users become more familiar with the mapping, the proposed recognition pipeline can adapt well to subtle physical variations in the micro-gesture signals and maintain stable input robustness. In other words, the initial disadvantage of the ergonomic layout lies primarily in learnability rather than in a lack of sensing reliability.

Overall, these findings suggest that SlideRing benefits from the physical contact mechanism of thumb-to-finger interaction. Unlike purely vision-based gesture input, which is often sensitive to occlusion, lighting, or unstable viewing conditions, frictional thumb contact on the finger surface provides passive proprioceptive and tactile feedback. This passive feedback reduces the need for constant visual verification, lowers visual-resource demand during input, and helps sustain eyes-free interaction in constrained mobile or immersive environments.

### 5.3. Subjective Workload

**Figure 9:**
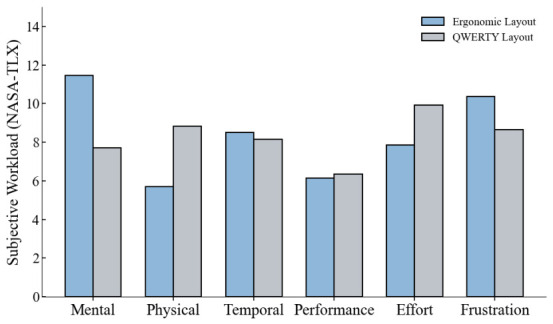
NASA-TLX workload comparison between the ergonomic and QWERTY-compatible SlideRing layouts across six subjective dimensions. Lower scores indicate lower perceived workload.

After the five-day online study, we used the NASA-TLX questionnaire [41] to evaluate participants’ subjective experience under the two layouts. The scale ranges from 1 to 20, with lower scores indicating lower perceived cognitive or physical demand. Figure 9 presents the workload distribution across the evaluated dimensions.

The subjective ratings reveal a clear tradeoff between physical and cognitive burden. To assess the statistical significance of these differences, we analyzed the NASA-TLX ratings using repeated-measures comparisons across participants.

From the perspective of physical workload, the ergonomic layout shows a clear biomechanical advantage. It yields significantly lower ratings for physical demand (M=5.71, SD=1.45) than the QWERTY-compatible layout (M=8.84, SD=1.73; $F_{1,11}=18.66$, p<0.01). A similar effect is observed for effort, where the ergonomic layout also scores lower (M=7.85, SD=1.62) than the QWERTY-compatible layout (M=9.92, SD=1.85; $F_{1,11}=9.92$, p<0.05). These results indicate that assigning high-frequency characters to short-span and low-energy motions effectively reduces local mechanical work of the thumb during prolonged use. More broadly, both layouts receive relatively low physical-load scores, which subjectively confirms that this micro-gesture paradigm avoids the pronounced arm fatigue often associated with suspended mid-air VR interaction.

In contrast, the QWERTY-compatible layout shows a clear advantage in mental demand. Its mental-demand rating (M=7.72, SD=1.24) is significantly lower than that of the ergonomic layout (M=11.45, SD=1.88; $F_{1,11}=39.43$, p<0.001). This finding is consistent with post-study interviews: participants reported that the QWERTY-compatible mapping allowed them to naturally reuse prior keyboard knowledge without deliberately recalling where each letter had been reassigned. By contrast, the ergonomic layout required them to temporarily suppress established keyboard habits during early use, thereby consuming more cognitive resources.

Interestingly, the two layouts do not differ significantly in frustration, even though the QWERTY-compatible layout produces a slightly lower mean frustration score (M=8.65, SD=1.52) than the ergonomic layout (M=10.36, SD=1.95). This pattern suggests that both layouts share a common underlying interaction challenge: they compress a full 26-letter alphabet into a dense gesture space distributed over a limited finger surface. As a result, users in both conditions experience some initial uncertainty and anxiety about accidental activation. This observation also helps explain the elevated early TER values and the performance variability seen during the initial learning phase.

Taken together, the subjective and objective findings indicate that SlideRing offers good overall usability. The ergonomic layout is more suitable for prolonged immersive text-entry scenarios because it reduces physical fatigue and supports stronger long-term performance, whereas the QWERTY-compatible layout offers a more approachable entry point for lightweight text input in constrained environments because of its lower cognitive threshold.

## 6. Discussion

The findings of this study should be interpreted in the context of a broader limitation of current VR text entry: many existing techniques still rely heavily on stable line of sight, sufficient illumination, or visually guided pointing. In contrast, SlideRing is built on local inertial sensing and thumb-to-finger physical contact. This design reduces reliance on camera visibility and therefore indicates potential suitability for constrained VR situations, such as when users rest their hands near the torso, type outside the effective tracking volume, or interact in mobile settings where visual pipelines become unstable.

To contextualize the observed entry speed, the final SlideRing performance reported in this study (15.75 WPM for the ergonomic layout and 15.25 WPM for the QWERTY-compatible layout) is comparable to several compact wearable or thumb-to-finger systems. For example, PinchType uses thumb-to-fingertip pinches with language-model disambiguation and reports 12.54 WPM, DRG-Keyboard uses dual IMU rings for subtle fingertip gesture typing and reports 12.9 WPM, and WrisText uses one-handed wrist gestures on a smartwatch and reaches 15.2 WPM by the final session [15,18,26]. Some visually guided or on-body keyboard methods can reach higher speeds; for instance, OnArmQWERTY projects a QWERTY keyboard onto the arm and reports 20.18 WPM for palm-based tapping [36]. However, such methods often rely on a larger visible keyboard, optical tracking, projected surfaces, or more constrained postures. SlideRing is therefore positioned not as the fastest universal text-entry technique, but as a minimal-wear and low-visual-demand alternative for constrained VR settings.

From a system-design perspective, the results also show that sensing performance alone is not sufficient; the keyboard mapping strategy plays an equally important role in shaping practical usability. The QWERTY-compatible layout offers a clear onboarding benefit because it reuses pre-existing keyboard knowledge and lowers the cognitive barrier for initial adoption. The ergonomic layout, in contrast, better supports long-term use because it maps frequent characters to biomechanically inexpensive gestures. The observed crossover between short-term learnability and long-term efficiency suggests that future VR text-entry systems may benefit from adaptive or personalized layout strategies rather than relying on a single fixed mapping for all users and all stages of learning.

Another important implication concerns the role of passive tactile feedback in immersive input. Unlike free-space mid-air typing, thumb contact on the finger surface provides a stable physical reference, frictional guidance, and proprioceptive confirmation. This self-haptic support helps reduce the need for continuous visual verification and also mitigates the arm fatigue commonly associated with suspended mid-air interaction [9,10]. At the same time, the remaining recognition errors indicate a specific technical bottleneck: the main ambiguity lies not in distinguishing taps from slides, but in separating short and long slides within the same direction. This suggests that future improvements should focus on better user-specific calibration, adaptive movement thresholds, and richer feedback mechanisms for motion extent.

This study also has several limitations. First, the participant sample is relatively modest and mainly consists of young university users, which may limit generalizability to broader populations. Second, although the sensing nodes are lightweight and wearable, the current prototype still performs model inference on a laptop rather than entirely on embedded hardware. Third, the present evaluation focuses on alphabetic English text entry and does not yet address multilingual input or more complex editing tasks. Future work should therefore examine larger and more diverse participant groups, fully on-device inference pipelines, and the extension of the mapping strategy to richer language and interaction settings. In particular, although Space, Backspace/Delete, and Enter/Confirm were handled through auxiliary non-contact thumb swipes during the experiment, these control gestures were not included in the trained contact-gesture classifier. The current study did not systematically evaluate punctuation, digits, or a full secondary symbol layer. Future work should integrate these remaining symbol and editing functions into a unified recognition framework and evaluate their learnability, robustness, and effect on text-entry efficiency. Finally, the present evaluation was conducted under controlled seated conditions. Although SlideRing is designed to reduce dependence on optical hand tracking and continuous visual attention, we did not systematically test low-light, occluded, out-of-view, mobile, or other challenging VR conditions. Future work should evaluate the system under these constrained scenarios to verify its robustness beyond comfortable laboratory settings.

## 7. Conclusions

In this work, we presented SlideRing, a dual-thumb IMU-based text-entry technique designed for VR scenarios in which purely vision-based hand interaction may be unreliable, visually demanding, or difficult to sustain. By anchoring input to thumb-to-finger contact, SlideRing transforms the finger surface into a compact and proprioceptively grounded micro-gesture space, and combines this interaction design with a dual-stream recognition model and two complementary keyboard layouts optimized for long-term movement economy and immediate learnability, respectively. In offline and online evaluations, the system achieved 96.5% target-finger accuracy and 94.2% action-type accuracy and demonstrated clear usability benefits in a five-day text-entry study: the QWERTY-compatible layout provided faster initial adoption and lower mental demand, whereas the ergonomic layout delivered better long-term performance and lower physical fatigue after practice. Overall, these findings indicate that lightweight inertial thumb-to-finger input has the potential to provide a robust, practical, and low-burden text-entry solution for constrained VR environments, while offering a promising path toward more adaptive and fatigue-aware immersive interaction.

## Figures and Tables

**Table 1:** SlideRing action space and relative physical effort.

Action	Description	Mechanical Characteristic	Effort
Tap	Brief contact on the target finger segment	Natural thumb flexion with clear tactile confirmation	Lowest
Short inward slide	Slide toward the palm over a short distance	Follows natural thumb adduction	Low
Short outward slide	Slide toward the fingertip over a short distance	Requires active thumb abduction	Medium
Long inward slide	Longer inward slide toward the palm	Larger travel with moderate muscular work	High
Long outward slide	Longer outward slide toward the fingertip	Largest travel and highest abduction torque	Highest

**Table 2:** Density-aware degradation rules for the QWERTY-compatible layout.

Density	Assigned Action Set	Mapping Logic	Typical Case
N=5	Tap + short inward/outward + long inward/outward	Full-capacity mode. Tap acts as the center anchor, short slides map to near neighbors, and long slides map to far neighbors.	Index-finger zones such as Y, U, I, O, P
N=4	Short inward/outward + long inward/outward	Anti-false-trigger mode. Tap is removed so that the resting position remains neutral and activation requires an explicit slide.	Middle-finger zones such as H, J, K, L
N=3	Tap + long inward/outward	High-tolerance mode. Short slides are removed, leaving the easiest central action and the most separable long-travel actions.	Ring-finger zones such as B, N, M
N=2	Long inward/outward	Binary mode for highly sparse regions with maximum separation between the two remaining choices.	Functional or edge zones
N=1	Tap	Confirmation mode with a single stable trigger.	Isolated keys

**Table 3:** Longitudinal text-entry performance across five practice days. Values are reported as mean ± SD.

Day	QWERTY WPM	Ergonomic WPM	QWERTY TER (%)	Ergonomic TER (%)
1	10.55 ± 1.23	7.43 ± 1.84	8.61 ± 1.45	13.42 ± 2.61
2	12.45 ± 1.05	11.15 ± 1.62	7.34 ± 1.32	11.73 ± 1.95
3	14.21 ± 0.88	12.88 ± 1.35	6.78 ± 1.05	8.35 ± 1.63
4	14.81 ± 0.94	14.67 ± 1.12	5.82 ± 1.12	7.21 ± 1.24
5	15.25 ± 0.72	15.75 ± 0.89	5.61 ± 0.86	6.14 ± 0.91

## Data Availability

The data presented in this study are available from the corresponding author upon reasonable request.
